# A New Maritime Moving Target Detection and Tracking Method for Airborne Forward-looking Scanning Radar

**DOI:** 10.3390/s19071586

**Published:** 2019-04-02

**Authors:** Weibo Huo, Jifang Pei, Yulin Huang, Qian Zhang, Jianyu Yang

**Affiliations:** School of Communication and Information Engineering, University of Electronic Science and Technology of China, Chengdu 611731, China; cqzhangq92@163.com (Q.Z.); jyyang@uestc.edu.cn (J.Y.)

**Keywords:** forward-looking scanning radar, sea clutter, track-before-detect, particle filter, spectral residual

## Abstract

Maritime moving target detection and tracking through particle filter based track-before-detect (PF-TBD) has significant practical value for airborne forward-looking scanning radar. However, villainous weather and surging of ocean waves make it extremely difficult to accurately obtain a statistical model for sea clutter. As the likelihood ratio calculation in PF-TBD is dependent on the distribution of the clutter, the performance of traditional distribution-based PF-TBD seriously declines. To resolve these difficulties, this paper proposes a new target detection and tracking method, named spectral-residual-binary-entropy-based PF-TBD (SRBE-PF-TBD), which is independent from the prior knowledge of sea clutter. In the proposed method, the likelihood ratio calculation is implemented by first extracting the spectral residual of the input image to obtain the saliency map, and then constructing likelihood ratio through a binarization processing and information entropy calculation. Simulation results show that the proposed method had superior performance of maritime moving target detection and tracking.

## 1. Introduction

The rapid development of global trade has greatly promoted the demand for marine transport and tourism, which also increases marine accidents [1,2,3]. Therefore, marine search and rescue has become an important research issue and receives a lot of attention in recent years [4,5]. The primary task of marine search and rescue is to discover the target under complex sea condition quickly and accurately, especially small targets, such as lifeboats carrying survivors. The airborne forward-looking scanning radar is a main detection means for marine search and rescue because of its all-weather and all-time capacity [6,7,8,9]. Through the sequential scanning of radar, multi-frame data can be quickly acquired and the maritime target in the observation area can be monitored in real time.

In general, fine weather condition and calm sea surface benefit target detection and tracking because of the high signal clutter ratio (SCR) of the radar echo. In this situation, the target can be effectively detected by traditional constant false alarm rate (CFAR) technologies [10,11,12], and then the trajectory can be estimated through the tracking filter [13,14]. However, villainous weather and surging of ocean waves will lead to heavy-tailed clutter and fluctuation of target scattering. In addition to the weak scattering of the target, the SCR will become quite low. In this case, traditional detection and tracking method will generate massive false alarms, making it hard to detect the true target [15].

Different from the traditional detection and tracking method, the TBD method utilizes the motion characteristics of the target and processes consecutive scans jointly to realize the fine detection and tracking of low SCR targets. Therefore, the TBD method has drawn extensive attention [16]. TBD includes batch methods, such as dynamic programming [17] and Hough transform [18], and recursive methods based on the Bayesian approach, such as particle filter (PF) [19]. Because PF can effectively deal with nonlinear non-Gaussian problems, it adapts to the target detection and tracking under sea scene. The earliest use of the PF-TBD method in radar system is presented in [20]. Subsequently, Rutten et al. proposed a more efficient implementation method of PF-TBD [21], and analyzed the characteristics of the method in detail [22]. Recently, PF-TBD is applied in different radar systems, such as over-the-horizon radar (OTHR) [23], passive radars [24], SAR [25] and multi-station system [26]. However, these studies consider the Rayleigh clutter distribution, which is only applicable to low resolution radars or low sea conditions. When the high-resolution radar observes the sea surface at a small grazing angle, the clutter distribution presents a significant non-Gaussian characteristic [27]. In [28], the PF-TBD method based on K-distribution adapts to the non-Gaussian characteristic of the sea clutter amplitude, and the calculation of likelihood ratio is deduced in detail. However, due to the complexity of the K-distribution, the analytical solution of the likelihood ratio calculation cannot be obtained, and this method must be resolved by a time-consuming numerical integration operation. This is a great challenge for real-time demand in applications.

Moreover, the distribution-based methods need to assume the prior distribution of clutter. However, the complex ocean dynamics mechanism gives the sea surface non-stationary characteristic [29], which means it is difficult to accurately model the sea surface with a hypothetical distribution model. To reduce the dependence on the prior knowledge of clutter, the saliency detection approach is proposed based on visual saliency principle [30,31,32]. A fast saliency detection approach proposed in [33] obtains the saliency map by analyzing the log-spectrum of an input image and extracting the spectral residuals of the image in the frequency domain, and then realize target detection.

Inspired by the saliency detection approach, this paper proposes a new target detection and tracking method, named spectral-residual-binary-entropy-based PF-TBD (SRBE-PF-TBD), which is used to detect and track maritime moving target for airborne forward-looking scanning radar. In the proposed method, the likelihood ratio calculation is implemented by first extracting the spectral residual of the input image to obtain the saliency map, and then constructing likelihood ratio through a binarization processing and information entropy calculation.

The proposed SRBE-PF-TBD method does not require prior knowledge of the clutter, enhancing the adaptability and robustness. The effectiveness and performance of the proposed method are verified by simulation experiments based on the simulated data, which are obtained by a Swerling I target and the K-distributed clutter.

This paper is arranged as follows. Section 2 introduces the target dynamic model and forward-looking scanning radar measurement model. Section 3 shows the proposed SRBE-PF-TBD method. Section 4 presents the simulation results and performance analysis. Section 5 gives the conclusions.

## 2. Models

In this section, the target dynamic model and the airborne forward-looking scanning radar measurement model are given, which are used in this paper.

### 2.1. Target Dynamic Model

Considering the maritime target moving in a two-dimensional plane, the state vector of the target in frame *k* is represented as $x^{k}={\left[x^{k},{\dot{x}}^{k},y^{k},{\dot{y}}^{k}\right]}^{T}$, where $\left(x^{k},y^{k}\right)$ is the position of the target and $\left({\dot{x}}^{k},{\dot{y}}^{k}\right)$ denotes the velocity along *x*-axis and *y*-axis, respectively. Assuming a constant-velocity process model is used, the evolution of the target state can be written as

(1) $$x^{k+1}=Fx^{k}+w^{k}$$

where

(2) $$F=\left[\begin{array}{cccc} 1 & \tau & 0 & 0\\ 0 & 1 & 0 & 0\\ 0 & 0 & 1 & \tau \\ 0 & 0 & 0 & 1 \end{array}\right]$$

τ is the frame sampling period, and $w^{k}$ represents the process noise, which obeys Gaussian distribution with mean zero and covariance Q:

(3) $$Q=q\times \left[\begin{array}{cccc} \tau^{3}/3 & \tau^{2}/2 & 0 & 0\\ \tau^{2}/2 & \tau & 0 & 0\\ 0 & 0 & \tau^{3}/3 & \tau^{2}/2\\ 0 & 0 & \tau^{2}/2 & \tau \end{array}\right]$$

where *q* is the process noise coefficient [34].

**Figure 1:**
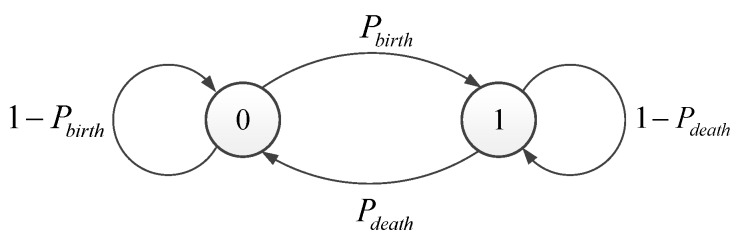
Diagram of state transition.

To indicate the existence or non-existence of target in the observation area, the random variable $\varepsilon^{k}$ is introduced as the target existence state variable and $\varepsilon^{k}\in \left\{0,1\right\}$, where $\;\varepsilon^{k}=1$ means there is a target in the observation area in frame *k* and vice versa. The above process can be modeled as a two-state Markov chain, as shown in Figure 1.

In Figure 1, $P_{birth}$ is the probability of target birth and $P_{death}$ is the probability of target death, which are respectively equal to $P\left(\varepsilon^{k}=1|\varepsilon^{k-1}=0\right)$ and $P\left(\varepsilon^{k}=0|\varepsilon^{k-1}=1\right)$. Thus, the two state Markov chain can be defined as

(4) $$\Pi =\left[\begin{array}{cc} 1-P_{birth} & P_{birth}\\ P_{death} & 1-P_{death} \end{array}\right]$$

### 2.2. Airborne Forward-Looking Scanning Radar Measurement Model

**Figure 2:**
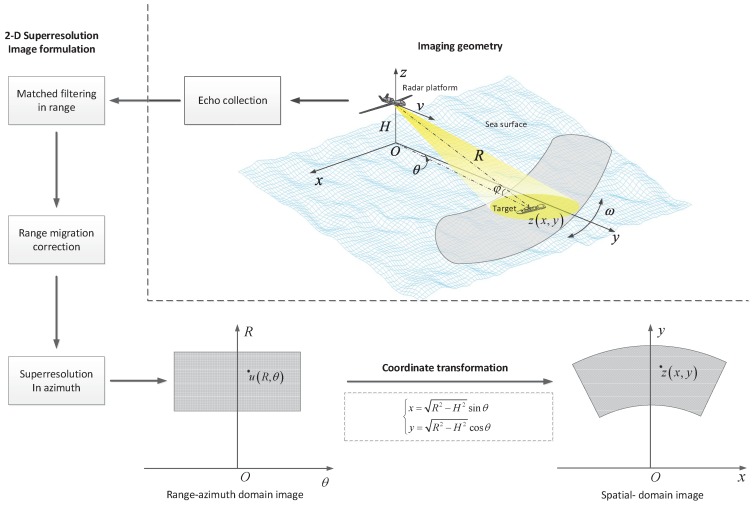
Geometry illustration for forward-looking scanning radar and the flowchart of imaging processing.

The geometry illustration for airborne forward-looking scanning radar is shown in Figure 2. For the common overlapping area of multi-frame images, the observation area $\Omega^{k}$ of frame *k* can be represented as

(5) $$\Omega^{k}=\left[\begin{array}{cccc} \sigma_{x_{1},y_{1}}^{k} & \sigma_{x_{1},y_{2}}^{k} & \cdots & \sigma_{x_{1},y_{N_{y}}}^{k}\\ \sigma_{x_{2},y_{1}}^{k} & \sigma_{x_{2},y_{2}}^{k} & \cdots & \sigma_{x_{2},y_{N_{y}}}^{k}\\ \vdots & \vdots & \ddots & \vdots \\ \sigma_{x_{N_{x}},y_{1}}^{k} & \sigma_{x_{N_{x}},y_{2}}^{k} & \cdots & \sigma_{x_{N_{x}},y_{N_{y}}}^{k} \end{array}\right]$$

where $\sigma_{x_{m},y_{n}}^{k}\left(m=1,2,\cdots ,N_{x},n=1,2,\cdots ,N_{y}\right)$ is the scattering coefficient of the point $\left(x_{m},y_{n},0\right)$ in the observation area, and $N_{x}$ and $N_{y}$ are the numbers of resolution cells along *x*-axis and *y*-axis, respectively. Assume the airplane is moving along *y*-axis at speed *v*, the waveform of transmitted signal is $s\left(\eta \right)$, where η is the fast time, the scanning speed of radar beam is ω and the antenna pattern is $w_{a}\left(t\right)$. At slow time *t*, the coordinate of radar is set as $\left(0,vt,H\right)$, and then the echo signal of the observation area in forward-looking scanning radar can be expressed as

(6) $$u^{k}\left(\eta ,t\right)=\sum_{m,n}\sigma_{x_{m},y_{n}}^{k}w_{a}\left(t\right)s\left(\eta -\frac{2R_{x_{m},y_{n}}\left(t\right)}{c}\right)$$

where

(7) $$R_{x_{m},y_{n}}\left(t\right)=\sqrt{R_{x_{m},y_{n}}^{2}+{\left(vt\right)}^{2}-2R_{x_{m},y_{n}}vt\cos\theta_{x_{m},y_{n}}\cos\varphi }$$

shows the instantaneous slant range of the point $\left(x_{m},y_{n},0\right)$, while $R_{x_{m},y_{n}}$ is the initial slant range, $\theta_{x_{m},y_{n}}$ is the initial azimuth angle and φ is the grazing angle. In practice, due to the narrow azimuth beam, fast scanning speed and small grazing angle [7], the slant range can be approximated as

(8) $$R_{x_{m},y_{n}}\left(t\right)\approx R_{x_{m},y_{n}}-vt\cos\varphi$$

For the convenience of spatial data analysis, the time variable *t* and η can be represented by the spatial variables *R* and θ based on t=θ−θ0θ−θ0ωω and η=2R2Rcc, where $\theta_{0}$ is initial scanning angle of the antenna. After the processing of matched filtering and range immigration correcting along the range dimension, the echo can be modeled as a convolution of the scattering coefficients and the two-dimensional system function [6], which is

(9) $$u^{k}\left(R,\theta \right)=\sum_{m,n}{\hat{\sigma }}_{x_{m},y_{n}}^{k}\delta \left(R-R_{x_{m},y_{n}},\theta -\theta_{x_{m},y_{n}}\right)⊗h\left(R,\theta \right)$$

where

(10) $${\hat{\sigma }}_{x_{m},y_{n}}^{k}=\sigma_{x_{m},y_{n}}^{k}\cdot \exp\left(-j\frac{4\pi }{\lambda }R_{x_{m},y_{n}}\left(t\right)\right)$$

and $\delta \left(R-R_{x_{m},y_{n}},\theta -\theta_{x_{m},y_{n}}\right)$ is the Dirac function at $\left(R_{x_{m},y_{n}},\theta_{x_{m},y_{n}}\right)$. $h\left(R,\theta \right)$ is the two-dimensional system function, which can be written as

(11) $$h\left(R,\theta \right)=w_{a}\left(\theta \right)\mathrm{sinc}\left(\frac{2B}{c}R\right)$$

where *B* is the bandwidth of the transmitted signal.

In azimuth, the resolution of radar image can be significantly enhanced by super-resolution processing, contributing to an improved two-dimensional system function [35].

After coordinate transferring of Equation (9), the final image in spatial domain can be represented as

(12) $$z^{k}\left(x,y\right)=\sum_{m,n}{\hat{\sigma }}_{x_{m},y_{n}}^{k}\delta \left(x-x_{m},y-y_{n}\right)⊗h^{\prime }\left(x_{m},y_{n}\right)$$

where $h^{\prime }\left(x_{m},y_{n}\right)$ is the two-dimensional system function in spatial domain. The imaging procedure of the forward-looking scanning radar is given in Figure 2.

Assuming the size of resolution cell in the observation area is $\Delta_{x}\times \Delta_{y}$, the measurement data in frame *k* can be expressed as

(13) $$z^{k}=\left\{z^{k}\left(m,n\right):m=1,2,\cdots ,N_{x},n=1,2,\cdots ,N_{y}\right\}$$

The hypothesis of the existence or non-existence of a target in resolution cell $\left(m,n\right)$ can be defined as:

(14) $$z^{k}\left(m,n\right)=\left\{\begin{array}{ll} \left|I^{k}\exp\left(j\phi^{k}\right)\cdot h^{\prime }\left(x_{m},y_{n}\right)+\varsigma^{k}\left(m,n\right)\right| & \varepsilon^{k}=1\\ \left|\varsigma^{k}\left(m,n\right)\right| & \varepsilon^{k}=0 \end{array}\right.$$

where $I^{k}$ is the amplitude of target, $\phi^{k}$ is the random phase of target, and $\varsigma^{k}\left(m,n\right)$ is the amplitude distribution of sea clutter, which is related with the sea condition and parameters of the surroundings.

## 3. The Proposed Method

In this section, the proposed SRBE-PF-TBD method is specified. The PF-TBD used in the proposed method is based on Bayesian recursion, which is derived in detail in [21]. In the PF-TBD, the particles are divided into two parts: the continuing particles, which represent the continuing presence of the target, and the birth particles, which represent the newly appearing target. By using the likelihood ratio to update the mixing weights of the two part particles, these particles can be mixed into a large set, and then the target state is estimated by the large set of particles.

Therefore, the likelihood ratio calculation plays an import role in the PF-TBD. As described above, the traditional distribution-based likelihood ratio calculation is infeasible in the situation of non-stationary sea surface. The proposed method implements likelihood ratio calculation from the perspective of image information, which is independent from the prior knowledge of clutter. Based on information theory, the information of an image can be decomposed into two parts: the prior knowledge and the innovation [33]. The prior knowledge part represents the statistical invariant properties of the environment, which can be considered as the redundant information and should be suppressed for target detection. The innovation part is the novel part, which contains information about target. Inspired by this rationale, the proposed method uses spectral residual calculation to remove the redundant information, and then the saliency map can be obtained. Finally, the likelihood ratio expression is constructed through a binary processing and information entropy calculation, which is named as SRBE likelihood ratio.

Based on the above, this section first presents the calculation of spectral residual binary entropy (SRBE) likelihood ratio, and then shows the implementation of the proposed SRBE-PF-TBD method.

### 3.1. SRBE Likelihood Ratio Calculation

Given the airborne forward-looking scanning radar image $z^{k}\left(m,n\right)$, $m=1,2,\cdots ,N_{x},n=1,2,\cdots ,N_{y}$ of frame *k*. The Fourier spectrum of the image can be calculated by

(15) $$Z^{k}\left(f_{m},f_{n}\right)=F\left[z^{k}\left(m,n\right)\right]$$

where F[·] means the operation of two-dimensional Fourier transform, and the corresponding amplitude spectrum and phase spectrum can be denoted as

(16) $$A^{k}\left(f_{m},f_{n}\right)=A\left[Z^{k}\left(f_{m},f_{n}\right)\right]$$

(17) $$P^{k}\left(f_{m},f_{n}\right)=P\left[Z^{k}\left(f_{m},f_{n}\right)\right]$$

where $A\left[\cdot \right]$ means getting the amplitude of the input, while $P\left[\cdot \right]$ obtains the phase. Then, the log spectrum of the image amplitude can be expressed as

(18) $$G^{k}\left(f_{m},f_{n}\right)=\ln\left[A^{k}\left(f_{m},f_{n}\right)\right]$$

Thus, the spectral residual can be obtained by

(19) $$R^{k}\left(f_{m},f_{n}\right)=G^{k}\left(f_{m},f_{n}\right)-\chi_{r}\left(f_{m},f_{n}\right)⊗G^{k}\left(f_{m},f_{n}\right)$$

where $\chi_{r}\left(f_{m},f_{n}\right)$ is a local mean filter, and defined by a r×r matrix:

(20) $$\chi_{r}\left(f_{m},f_{n}\right)=\frac{1}{r^{2}}\left[\begin{array}{cccc} 1 & 1 & \cdots & 1\\ 1 & 1 & \cdots & 1\\ \vdots & \vdots & \ddots & \vdots \\ 1 & 1 & \cdots & 1 \end{array}\right]$$

By performing two-dimensional inverse Fourier transform and Gaussian filtering, the saliency map in spatial domain can be expressed as

(21) $$S^{k}\left(m,n\right)=g\left(m,n\right)⊗F^{-1}{\left[\exp\left(R^{k}\left(f_{m},f_{n}\right)+P^{k}\left(f_{m},f_{n}\right)\right)\right]}^{2}$$

where $F^{-1}\left[\cdot \right]$ means the operation of two-dimensional inverse Fourier transform. The Gaussian filer $g\left(m,n\right)$ is defined by

(22) $$g\left(m,n\right)=\frac{1}{2\pi \gamma^{2}}\exp\left(-\frac{m^{2}+n^{2}}{2\gamma^{2}}\right)$$

where γ is the filter parameter.

Then, the normalized saliency map can be obtained by

(23) $${\bar{S}}^{k}\left(m,n\right)=\frac{S^{k}\left(m,n\right)}{\max\left[S^{k}\left(m,n\right)\right]}$$

By a given binarization threshold $T_{B}$, the normalized saliency map is binarized and the corresponding binary image $B^{k}$ is generated, which indicates the potential target area. Then, the information entropy of $B^{k}$ is calculated by

(24) $$H^{k}=-p_{0}^{k}\cdot \log_{2}\left(p_{0}^{k}\right)-p_{1}^{k}\cdot \log_{2}\left(p_{1}^{k}\right)$$

where $p_{0}$ and $p_{1}$, respectively, indicate the probabilities of the pixel value 0 and 1 in the binary image $B^{k}$.

The final SRBE likelihood ratio can be written as

(25) $$L_{SRBE}\left(z^{k}|\varepsilon^{k}\right)=\frac{p_{1}^{k}}{p_{0}^{k}}\cdot \exp\left(\frac{{\bar{S}}^{k}\left(m,n\right)}{H^{k}}\right)$$

For the target with state $x^{k}$, the coordinate of the corresponding cell can be calculated by

(26) $$\left\{\begin{array}{c} m_{x_{k}}=\left\lfloor \frac{x^{k}}{\Delta_{x}}\right\rfloor \\ n_{x_{k}}=\left\lfloor \frac{y^{k}}{\Delta_{y}}\right\rfloor \end{array}\right.$$

where $\left\lfloor \cdot \right\rfloor$ denotes the operation of rounding down. The SRBE likelihood ratio corresponding to $x^{k}$ can be calculated by

(27) $$L_{SRBE}\left(z^{k}|x^{k},\varepsilon^{k}\right)=\frac{p_{1}^{k}}{p_{0}^{k}}\cdot \exp\left(\frac{{\bar{S}}^{k}\left(m_{x_{k}},n_{x_{k}}\right)}{H^{k}}\right)$$

**Figure 3:**
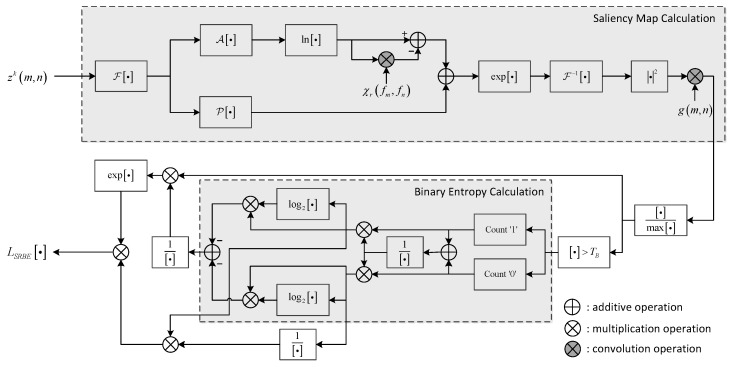
Flowchart of SRBE likelihood ratio calculation.

The flowchart of SRBE likelihood ratio calculation is shown in Figure 3.

As shown in Figure 3, the proposed SRBE method has two key operations: one is obtaining the saliency map of the radar image by the spectral residual method, and the other is calculating the binary entropy of the normalized saliency map. Because the maritime target is generally composed of metal material and has dihedral and trihedral structures, the scattering of the target is normally higher than the scattering of the sea clutter, meaning that the target is more salient than the sea clutter in the radar image. Therefore, the saliency map can effectively indicate the potential target regions. Through the information theory, the binary entropy of the saliency map can be used to measure the cluster-degree of the saliency map. The larger the entropy value is, the smaller the cluster-degree is, indicating that the saliency map is more likely caused by the sea clutter scattering. Contrarily, the lower entropy value represents the larger cluster-degree, which means the higher probability of target existence. Therefore, the binary entropy of the saliency map can effectively indicate the confidence of target existence. Based on the above analysis, the SRBE likelihood is constructed as Equation (27) to update the particles, which utilizes the information of the potential target regions and the confidence of target existence in the radar image.

### 3.2. Implementation of SRBE-PF-TBD

By incorporating the SRBE likelihood ratio into the PF-TBD, the specific implementation of the proposed SRBE-PF-TBD is described as follows.

1. Calculation of birth density
  A set of $N_{b}$ birth particles is drawn from the proposal density, whose positions are uniformly distributed within some highest measurements [22]. The sampled birth particles for frame *k* can be represented as
  (28) $$x_{(b)i}^{k}∼q\left(x_{\left(b\right)}^{k}|\varepsilon^{k}=1,\varepsilon^{k-1}=0,z^{k}\right),i\in \left\{1,2,\ldots ,N_{b}\right\}$$
  where $q\left(\cdot \right)$ is the proposal density, and the symbol “∼” means the operation of drawing samples from the proposal density.
  Based on the likelihood ratio proposed in Equation (27), the non-normalized weights of these birth particles can be calculated by
  (29) $$\omega_{\left(b\right)i}^{k}=\frac{L_{SRBE}\left(z^{k}|x_{\left(b\right)i}^{k},\varepsilon^{k}=1\right)p\left(x_{\left(b\right)i}^{k}|\varepsilon^{k}=1,\varepsilon^{k-1}=0\right)}{N_{b}q\left(x_{\left(b\right)i}^{k}|\varepsilon^{k}=1,\varepsilon^{k-1}=0,z^{k}\right)}$$
  where the prior probability density $p\left(x_{\left(b\right)i}^{k}|\varepsilon^{k}=1,\varepsilon^{k-1}=0\right)$ is a uniform distribution. Then, the normalized weights can be obtained by
  (30) $${\tilde{\omega }}_{\left(b\right)i}^{k}=\frac{\omega_{\left(b\right)i}^{k}}{\sum_{i=1}^{N_{b}}\omega_{\left(b\right)i}^{k}}$$
2. Calculation of continuing density
  Since the auxiliary particle filter (APF) utilizes the information of measurement in the current frame, the knowledge of current observation is incorporated into the proposal mechanism, and particles are not moved blindly in the state space, making the current target state estimated with high reliability [36]. Considering these benefits, this paper adopts APF for continuing particle filtering.
  A set of $N_{c}$ continuing particles is drawn using the dynamic equation described in Equation (1), which can be expressed as
  (31) $$\mu_{(c)i}^{k}∼p\left(x_{\left(c\right)}^{k}|x_{\left(c\right)}^{k-1},\varepsilon^{k}=1,\varepsilon^{k-1}=1\right),i\in \left\{1,2,\ldots ,N_{c}\right\}$$
  where $p\left(\cdot \right)$ represents the transition density from frame k−1 to frame *k*.
  The non-normalized weights of these particles are calculated with the current measurements as below.
  (32) $$b_{(c)i}^{k}=\frac{1}{N_{c}}L_{SRBE}\left(z^{k}|\mu_{(c)i}^{k},\varepsilon^{k}=1\right)$$
  The normalized weights of continuing particles can be calculated by
  (33) $${\tilde{b}}_{\left(c\right)i}^{k}=\frac{b_{\left(c\right)i}^{k}}{\sum_{i=1}^{N_{c}}b_{\left(c\right)i}^{k}}$$
  By using weights ${\tilde{b}}_{\left(c\right)i}^{k}$, the particles ${\left\{x_{\left(c\right)i}^{k-1}\right\}}_{i=1}^{N_{c}}$ in frame k−1 are resampled to obtain ${\left\{x_{\left(c\right)i^{j}}^{k-1}\right\}}_{j=1}^{N_{c}}$, where ${\left\{i^{j}\right\}}_{j=1}^{N_{c}}$ denotes the index of particle $x_{\left(c\right)i}^{k-1}$ in frame k−1.
  Then, particles for frame *k* by Equation (1) and the resampled continuing particles in frame k−1 are again drawn, which can be expressed as
  (34) $$x_{(c)j}^{k}∼p\left(x_{\left(c\right)}^{k}|x_{\left(c\right)i^{j}}^{k-1},\varepsilon^{k}=1,\varepsilon^{k-1}=1\right),j\in \left\{1,2,\ldots ,N_{c}\right\}$$
  The non-normalized weights of newly resampled particles can be calculated by
  (35) $$\omega_{(c)j}^{k}=\frac{L_{SRBE}\left(z^{k}|x_{(c)j}^{k},\varepsilon^{k}=1\right)}{L_{SRBE}\left(z^{k}|\mu_{\left(c\right)i^{j}}^{k},\varepsilon^{k}=1\right)}$$
  The normalized weights can be obtained by
  (36) $${\tilde{\omega }}_{\left(c\right)j}^{k}=\frac{\omega_{\left(c\right)j}^{k}}{\sum_{j=1}^{N_{c}}\omega_{\left(c\right)j}^{k}}$$
3. Calculation of mixing probabilities
  The mixing probability of birth density is calculated by
  (37) $$M_{b}=P_{birth}\left(1-p\left(\varepsilon^{k-1}=1|z^{1:k-1}\right)\right)\sum_{i=1}^{N_{b}}\omega_{\left(b\right)i}^{k}$$
  Similarly, the mixing probability of continuing density is calculated by
  (38) $$M_{c}=\left(1-P_{death}\right)p\left(\varepsilon^{k-1}=1|z^{1:k-1}\right)\sum_{i=1}^{N_{c}}\omega_{\left(c\right)i}^{k}$$
  Then, the normalized mixing probabilities can be expressed by
  (39) $${\tilde{M}}_{b}=\frac{M_{b}}{M_{b}+M_{c}}$$
  (40) $${\tilde{M}}_{c}=\frac{M_{c}}{M_{b}+M_{c}}$$
4. Calculation of scaled particle weights
  The particle weights are scaled according to the mixing probabilities, and the scaled particle weights of birth density and continuing density are obtained:
  (41) $${\hat{\omega }}_{\left(b\right)i}^{k}={\tilde{M}}_{b}{\tilde{\omega }}_{\left(b\right)i}^{k}$$
  (42) $${\hat{\omega }}_{\left(c\right)i}^{k}={\tilde{M}}_{c}{\tilde{\omega }}_{\left(c\right)i}^{k}$$
  Then, the particles of birth density and continuing density are combined into one large particle set:
  (43) $$\left\{\left(x_{\left(j\right)i}^{k},{\hat{\omega }}_{\left(j\right)i}^{k}\right)|i\in \left\{1,...,N_{j}\right\},j\in \left\{b,c\right\}\right\}$$
  The particles of the large set are resampled to obtain $N_{c}$ particles with uniform weights:
  (44) x^cik,11NcNc|i∈1,...,Nc
5. Calculation of probability of existence
  The probability of existence in frame *k* can be expressed as
  (45) $$\hat{p}\left(\varepsilon^{k}=1|z^{1:k}\right)=\frac{M_{b}+M_{c}}{M_{b}+M_{c}+P_{death}p\left(\varepsilon^{k-1}=1|z^{1:k-1}\right)+\left(1-P_{birth}\right)\left(1-p\left(\varepsilon^{k-1}=1|z^{1:k-1}\right)\right)}$$
6. Estimation of target state
  The detection threshold of existence is assumed as $P_{th}$. When $\hat{p}\left(\varepsilon^{k}=1|z^{1:k}\right)>P_{th}$, the hypothesis of target existence is accepted and the target state will be estimated by
  (46) $${\hat{x}}^{k}=\frac{1}{N_{c}}\sum_{i=1}^{N_{c}}{\hat{x}}_{\left(c\right)i}^{k}$$

## 4. Experimental Results

To verify the proposed method, simulation experiments of maritime moving target detection and tracking based on simulated data were conducted.

The simulated data of airborne forward-looking scanning radar consisted of 30 frames, whose frame sampling period was τ=1s, and per frame contained $N_{x}\times N_{y}=60\times 60$ bins. The target appeared in frame $K_{b}=6$, disappeared in frame $K_{d}=21$, and moved according to the dynamic equation (Equation (1)), while the process noise coefficient was q=0.001. The initial state of the target was set as $x^{0}={\left[\begin{array}{cccc} 12.2 & 1.2 & 15.1 & 0.8 \end{array}\right]}^{T}$.

There were 4000 particles, where the numbers used for birth density and continuing density were $N_{b}=2000$ and $N_{c}=2000$, respectively. The initial particle velocities were sampled uniformly from interval $\left[0,2\right]$, and the particle positions were uniformly distributed within the 200 highest measurements. The probabilities of birth and death are defined as $P_{birth}=P_{death}=0.05$, while the detection threshold was set as $P_{th}=0.5$. The binarization threshold $T_{B}$ was set to 0.5. All simulation results were averaged over 100 Monte Carlo trials.

### 4.1. Simulated Data

In practice, since airborne forward-looking scanning radar is used for long-range detection with small grazing angle, it is reasonable to model the sea clutter amplitude as K-distribution [29], which can be expressed as

(47) $$p\left(\varsigma^{k}\right)=\frac{2\beta }{\Gamma \left(\alpha \right)}{\left(\frac{\beta \varsigma^{k}}{2}\right)}^{\alpha }K_{\alpha -1}\left(\beta \varsigma^{k}\right)$$

where α is the shape parameter of the distribution, while β represents the scale parameter, and $K_{\alpha -1}\left(\cdot \right)$ is the modified second-kind Bessel function of order α−1.

Due to the movement of the ocean, the RCS of the maritime moving target varies between radar scans. Therefore, Swerling type I model was used to model the maritime moving target in our simulation [37]. The amplitude of the target can be written as

(48) $$p\left(I^{k}\right)=\frac{2I^{k}}{\bar{\sigma }}\exp\left(-\frac{{\left(I^{k}\right)}^{2}}{\bar{\sigma }}\right)$$

where $\bar{\sigma }$ denotes the mean squared target amplitude.

Thus, the SCR can be defined as

(49) $$\mathrm{SCR}=10\log_{10}\frac{\bar{\sigma }}{P_{clutter}}$$

where $P_{clutter}$ means the mean power of the clutter in frame *k* and can be expressed by

(50) $$P_{clutter}=\frac{4\alpha }{\beta^{2}}$$

### 4.2. Simulation Results

#### 4.2.1. Feasibility Analysis

**Figure 4:**
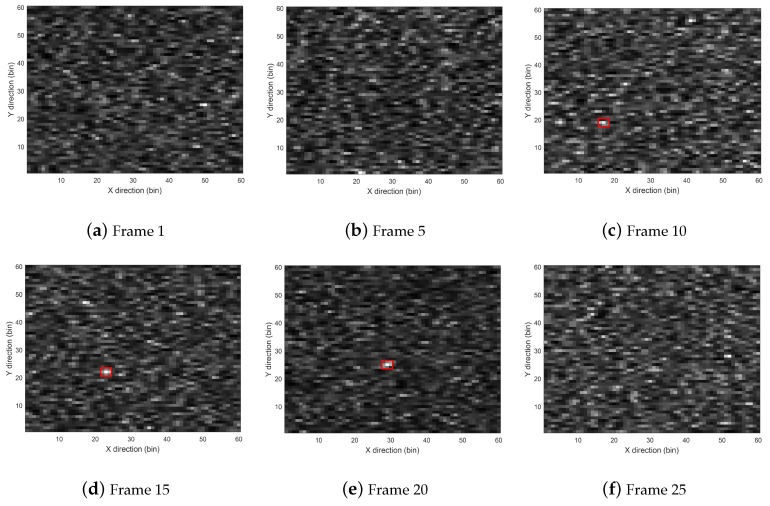
Sampled images at different frames.

The feasibility of the proposed method was demonstrated when SCR was set to 10dB. Figure 4 shows the radar images of Frames 1, 5, 10, 15, 20 and 25. The target exists in Frames 10, 15 and 20, and the positions of the target are marked by red rectangles in corresponding images. As shown in Figure 4, the target was difficult to distinguish from the clutter background.

**Figure 5:**
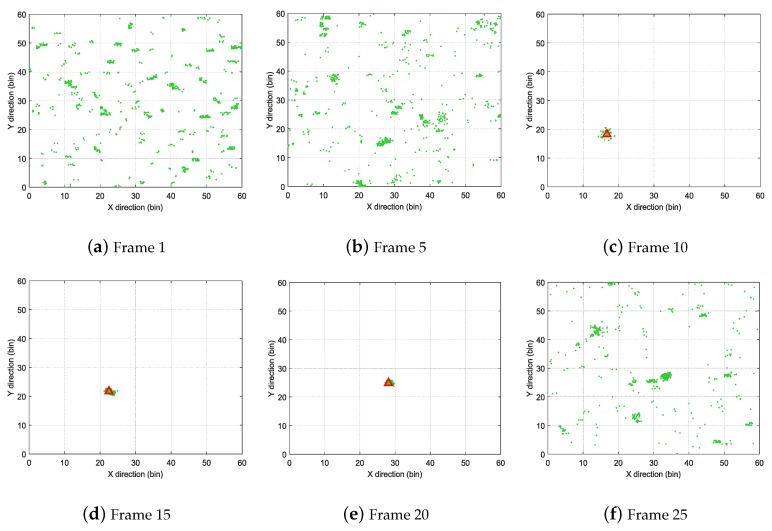
Distribution of particles corresponding with frames in Figure 4.

Figure 5 illustrates the distribution of particles calculated by the proposed SRBE-PF-TBD method, where the red triangles show the true positions of the target. When there was no target in Frames 1 and 5, particles were distributed according to the saliency map of the corresponding frame. In Frames 10, 15 and 20, where the target existed, the particles clustered around the target. When the target did not exist in Frame 25, the particles were distributed according to the saliency map again.

**Figure 6:**
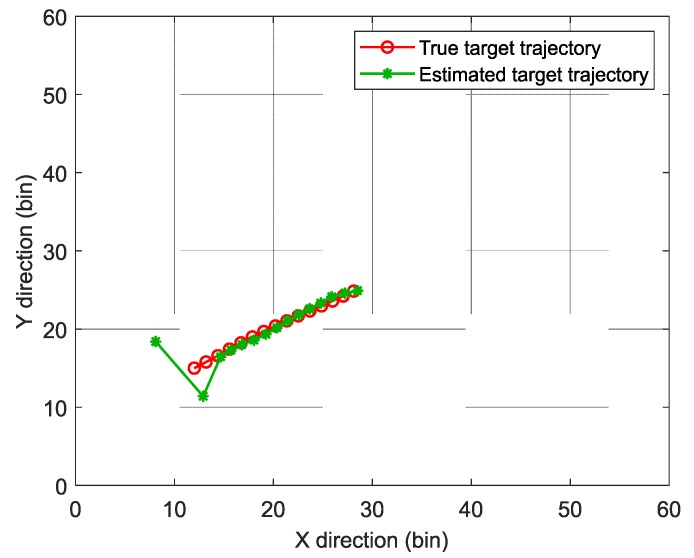
The estimation results of the proposed method.

The true target trajectory and the estimated trajectory are shown in Figure 6. In the first two frames where the target existed, the target positions were not well estimated. However, with the accumulation of frames, the SRBE-PF-TBD method tracked the target effectively, which verified the effectiveness of SRBE-PF-TBD method.

#### 4.2.2. Performance Evaluation

To analyze the performance of the proposed method, this section first gives the definition of the evaluation criteria [28,38,39], and then prsents the performance evaluation of the proposed method.

1. Root Mean Squared Error (RMSE) of Estimated Position
  The RMSE is calculated by
  (51) $$\mathrm{RMSE}\left(k\right)=\frac{1}{M}\sum_{i=1}^{M}\sqrt{{\left({\hat{x}}_{i}^{k}-x_{i}^{k}\right)}^{2}+{\left({\hat{y}}_{i}^{k}-y_{i}^{k}\right)}^{2}}\;\;\;k=K_{b},...,K_{d}-1$$
  where *M* is the number of Monte Carlo experiments, and $\left({\hat{x}}_{i}^{k},{\hat{y}}_{i}^{k}\right)$ and $\left(x_{i}^{k},y_{i}^{k}\right)$ denote the estimated position and true position of the target in the *i*th Monte Carlo experiment, respectively.
2. Average RMSE
  The average RMSE is calculated by
  (52) $$\bar{\mathrm{RMSE}}=\frac{1}{K_{\Delta }}\sum_{k=K_{b}}^{K_{d}-1}\bar{\mathrm{RMSE}}\left(k\right)$$
  where $K_{\Delta }=K_{d}-K_{b}$.
3. Detection Probability
  (53) $$P_{d}=\frac{1}{M}\sum_{i=1}^{M}\frac{K_{num}\left[\hat{p}\left(\varepsilon^{k}=1|z^{1:k}\right)>P_{th}\right]}{K_{\Delta }}\;\;\;k=K_{b},...,K_{d}-1$$
  where $K_{num}\left[\hat{p}\left(\varepsilon^{k}=1|z^{1:k}\right)>P_{th}\right]$ is the number of frames that satisfy the decision $\hat{p}\left(\varepsilon^{k}=1|z^{1:k}\right)>P_{th}$ in the *i*th Monte Carlo experiment.
4. False-alarm Probability
  (54) $$P_{fa}=\frac{1}{M}\sum_{i=1}^{M}\frac{K_{num}\left[\hat{p}\left(\varepsilon^{k}=1|z^{1:k}\right)>P_{th}\right]}{K-K_{\Delta }}\;\;\;k=\left\{1,...,K_{b}-1\right\}\cup \left\{K_{d},...,K\right\}$$
5. Earliest Effective Detection Frame
  (55) $$F_{e}=\frac{1}{M}\sum_{i=1}^{M}F_{i}$$
  where $F_{i}$ represents the frame number that the target is first detected in the *i*th Monte Carlo experiment.

**Figure 7:**
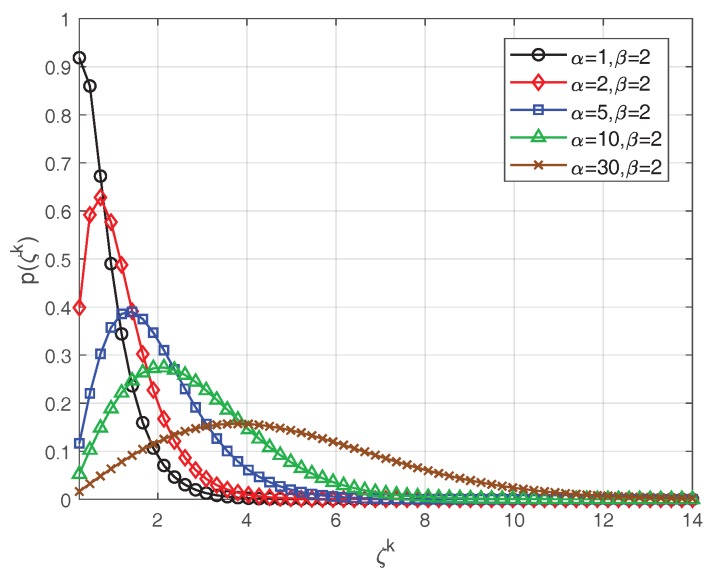
Probability density functions of K-distribution with different shape and scale parameters.

**Figure 8:**
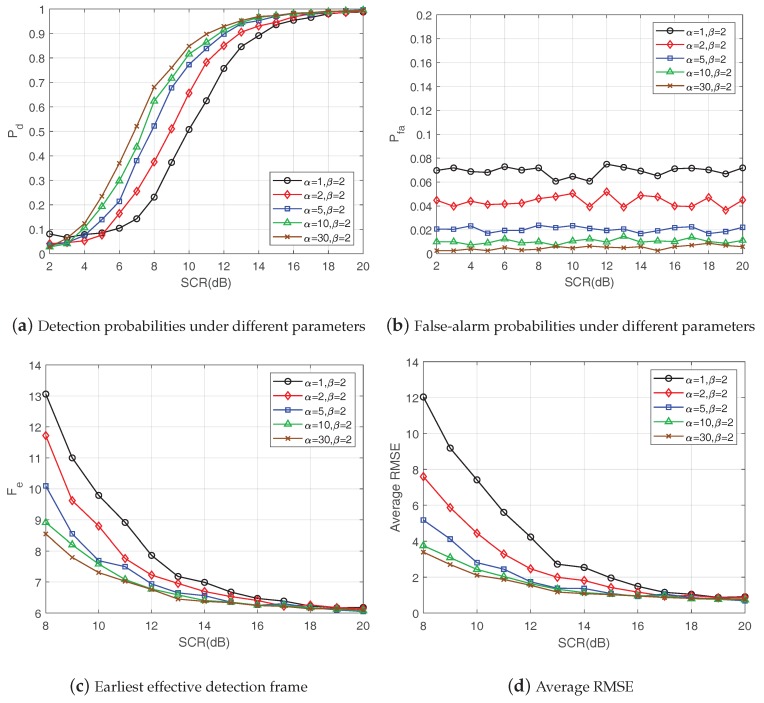
Performances under different K-distribution parameters.

Figure 7 shows the probability density functions of the K-distribution corresponding to five different sets of parameters, while Figure 8 demonstrates the performance of the proposed method under these different K-distribution parameters. Figure 8a–d, respectively, shows the detection probabilities, false-alarm probabilities, earliest effective detection frame and average RMSE of the proposed method with a fixed detection threshold under different SCRs. The results in Figure 8a,c,d show that, with the increase of SCR, the detection probability increased, while the earliest effective detection frame and the average RMSE declined. In the case of the same SCR, the proposed method performed better with larger shape parameters. As for the false-alarm probabilities in Figure 8b, the false-alarm probabilities basically remained consistent under different SCRs when the distribution parameters of the K-distribution were fixed. Meanwhile, the false alarm probability decreased when the shape parameter α became larger. These are reasonable because, when the shape parameter of the K-distribution increased, the heavy-tailed effect of K-distribution became weaker. One extreme case was that, when the shape parameter tended to infinity, the K-distribution degenerated into a Rayleigh distribution.

**Figure 9:**
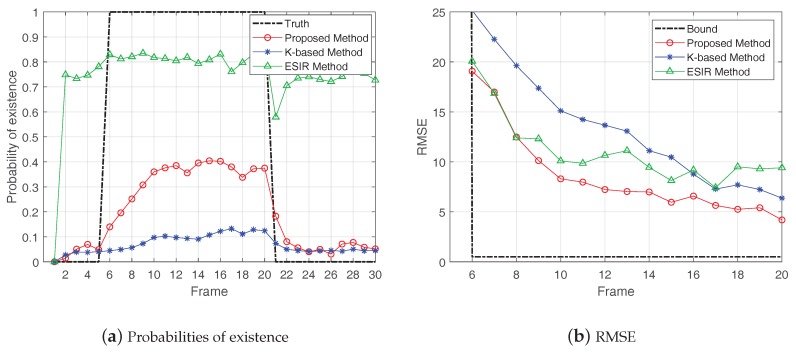
Probabilities of existence and Results of RMSE in case of SCR = 8 dB.

**Figure 10:**
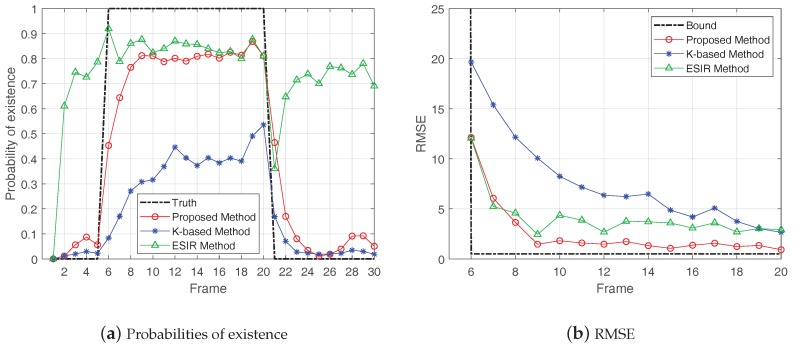
Probabilities of existence and Results of RMSE in case of SCR = 12 dB.

**Figure 11:**
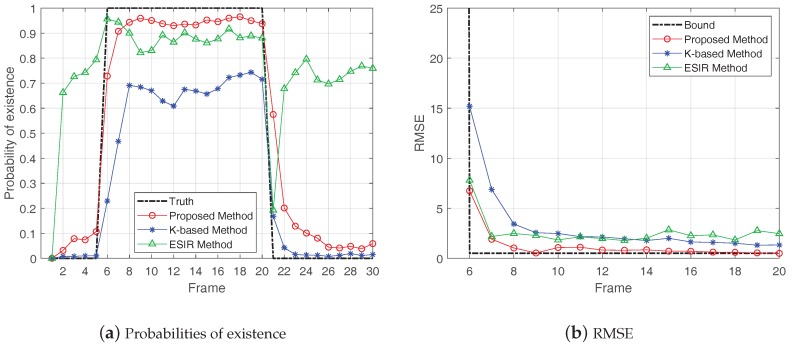
Probabilities of existence and Results of RMSE in case of SCR = 16 dB.

Then, the proposed method was compared with other two methods: the K-based method [28] and the ESIR method [21]. Figure 9, Figure 10 and Figure 11, respectively, show the probabilities of existence and RMSEs calculated by the three methods in cases of SCR = 8 dB, 12 dB and 16 dB. The shape parameter of K-distribution was set as α=2 and the scale parameter aws set as $\beta =2\sqrt{2}$, thus $P_{clutter}=1$.

In the results of probability of existence, the frames of true target existence are represented by the black dash line. From the results of probabilities of existence, the K-based method and the proposed method had better target existence estimation with higher SCR. In the case of the same SCR, the proposed method performed better than the K-based method. The ESIR method had the best estimation of target existence when there was a target. However, in the case of target non-existence, the ESIR method still gave a high probability of existence, representing a large false alarm. This is because the ESIR method is based on Rayleigh distribution, which will generate large false alarm when the clutter has the heavy-tailed feature.

In the results of RMSEs, the RMSE bound is illustrated by the black dash line. The results show that the RMSEs of these methods decreased with the increase of SCR, which means the estimated positions of the three methods gradually converged to the true position of the target with the accumulation of target existence time. In comparison, the estimation accuracy of the proposed method was superior to the other two methods.

**Figure 12:**
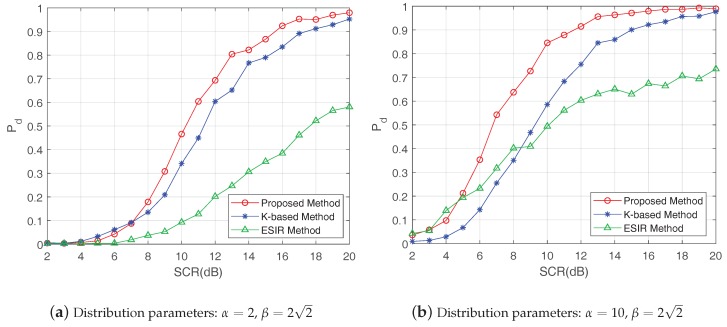
Detection probabilities of the three methods in case of *P_fa_* = 10^−2^.

Figure 12 shows the detection probabilities of the three methods under different SCRs in the case the false-alarm probability was equal to $10^{-2}$. In Figure 12a, the K-distribution parameters were set as α=2 and $\beta =2\sqrt{2}$. In this situation, the K-based method performed best when the SCR was below 8 dB, while the proposed method became superior to the other two methods with the increasing SCR. Meanwhile, it is worth noting that the detection probabilities of the three method were all smaller than 0.1 when the SCR was lower than 8 dB, indicating the inability to achieve reliable target detection in practical applications. In Figure 12b, the K-distribution parameters were α=10 and $\beta =2\sqrt{2}$, which means a smoother K-distributed clutter compared with Figure 12a. While the SCR was lower than 5 dB in Figure 12b, the detection probability of the ESIR method was slightly higher than the other two methods, which was attributed to the approximate Rayleigh distributed clutter in this case. When the SCR was beyond 5 dB, the proposed method had better detection performance than the other methods. Based on these results, it can be concluded that the proposed method can achieve better detection performance under more extensive clutter distribution parameters compared with the other two methods.

#### 4.2.3. Computational Efficiency

Computational efficiency is a key indicator for real-time processing. To compare the computational efficiency of the three methods, all experiments were done in MATLAB R2018a, while the computer CPU was Intel (R) Core (TM) i5-7500 3.40 GHz, with 16 GB RAM.

Each frame contained 60×60 bins, the number of Monte Carlo experiments was 300, and the average running time of single frame was calculated by

(56) $${\bar{t}}_{f}=\frac{1}{300}\sum_{i=1}^{300}t_{f}$$

where $t_{f}$ is the running time of single frame.

The computational consumption results are given in Table 1, which shows the average running time of the three methods with different numbers of particles. The results demonstrate that the proposed method and the ESIR method were obviously superior to the K-based method in term of running time. The K-based method calculated the likelihood ratio by a numerical solution of integral operation, causing a huge computational consumption. The computational efficiency of ESIR method was slightly higher than the proposed method, but, as shown in previous experimental results, the ESIR method only worked under the Rayleigh distribution clutter and caused high false alarm under the non-Gaussian clutter background.

All these experimental results demonstrate that the proposed method had fine detection and tracking performance and high computational efficiency.

## 5. Conclusions

In this paper, an effective maritime moving target detection and tracking method motivated by the human vision system is presented for airborne forward-looking scanning radar. The key idea of the proposed method is to use the saliency information of radar image to construct the SRBE likelihood ratio, which is independent from the prior knowledge of sea clutter. The proposed method effectively deals with the difficulty in target detection and tracking caused by non-Gaussian and non-stationary sea clutter. The performance of the proposed method was verified by Monte Carlo experiments using K-distribution clutter and Swerling type I target model. In the simulation experiments, the proposed method was evaluated under different K-distribution clutter parameters and compared with other two methods. The experimental results demonstrate that the proposed method had superior performance of target detection and tracking. Besides, the proposed method was computationally efficient and suitable for real-time application.

There are several continuing areas of research for improving the proposed method. The proposed method has been verified by simulated data, and future research will be to apply the proposed method to measured data. In addition, the extension of single-target situation to multi-target situation is the field to be researched.

## Figures and Tables

**Table 1:** Computational consumption.

Number of Particles	Proposed Method	ESIR Method	K-Based Method
4000	5.57 ms	5.51 ms	2.19 s
6000	8.79 ms	7.20 ms	3.27 s
8000	11.64 ms	9.06 ms	4.39 s
10,000	15.23 ms	11.46 ms	5.4 s
